# The Role of NMDA Receptors in the Effect of Purinergic P2X7 Receptor on Spontaneous Seizure Activity in WAG/Rij Rats With Genetic Absence Epilepsy

**DOI:** 10.3389/fnins.2020.00414

**Published:** 2020-05-06

**Authors:** Elif Doǧan, Hatice Aygün, Gökhan Arslan, Emil Rzayev, Bahattin Avcı, Mustafa Ayyıldız, Erdal Ağar

**Affiliations:** ^1^Department of Physiology, Faculty of Medicine, Ondokuz Mayıs University, Samsun, Turkey; ^2^Department of Physiology, Faculty of Medicine, Gaziosmanpasa University, Tokat, Turkey; ^3^Department of Clinical Biochemistry, Faculty of Medicine, Ondokuz Mayıs University, Samsun, Turkey

**Keywords:** absence, epilepsy, memantine, NMDA, oxidative, P2X7, stress, WAG/Rij

## Abstract

P2X7 receptors (P2X7Rs) are ATP sensitive cation channels and have been shown to be effective in various epilepsy models. Absence epilepsy is a type of idiopathic, generalized, non-convulsive epilepsy. Limited data exist on the role of P2X7Rs and no data has been reported regarding the interaction between P2X7Rs and glutamate receptor NMDA in absence epilepsy. Thus, this study was designed to investigate the role of P2X7 and NMDA receptors and their possible interaction in WAG/Rij rats with absence epilepsy. Permanent cannula and electrodes were placed on the skulls of the animals. After the healing period of the electrode and cannula implantation, ECoG recordings were obtained during 180 min before and after drug injections. P2X7R agonist BzATP, at doses of 50 μg and 100 μg (intracerebroventricular; i.c.v.) and antagonist A-438079, at doses of 20 μg and 40 μg (i.c.v.) were administered alone or prior to memantine (5 mg/kg, intraperitoneal; i.p.) injection. The total number (in every 20 min), the mean duration, and the amplitude of spike-wave discharges (SWDs) were calculated and compared. Rats were decapitated and the right and left hemisphere, cerebellum, and brainstem were separated for the measurements of the advanced oxidation protein product (AOPP), malondialdehyde (MDA), superoxide dismutase (SOD), glutathione (GSH), catalase (CAT), glutathione peroxide (GPx), and glutathione reductase (GR). BzATP and A-438079 did not alter measured SWDs parameters, whereas memantine reduced them, which is considered anticonvulsant. BzATP did not alter the anticonvulsant effect of memantine, while A-438079 decreased the effect of memantine. Administration of BzATP increased the levels of SOD and GR in cerebrum hemispheres. A-438079 did not alter any of the biochemical parameters. Memantine reduced the levels of MDA, GSH, and GR while increased the level of CAT in the cerebrum. Administration of BzATP before memantine abolished the effect of memantine on MDA levels. The evidence from this study suggests that P2X7Rs does not directly play a role in the formation of absence seizures. P2X7Rs agonist, reduced the antioxidant activity of memantine whereas agonist of P2X7Rs reduced the anticonvulsant action of memantine, suggesting a partial interaction between P2X7 and NMDA receptors in absence epilepsy model.

## Introduction

P2X7 receptors (P2X7Rs) are purinergic cation channels. They are sensitive to high concentrations of ATP in the extracellular space, and they have essential roles in inflammation (Burnstock and Knight, 2018). P2X7Rs could be permeable to small cations when they are stimulated within milliseconds. However, prolonged stimulation (within seconds) of P2X7Rs allows permeation by molecules with a mass of up to 900 Da, leading to the release of inflammatory cytokines and apoptosis (Burnstock and Knight, 2018). Stimulation of P2X7Rs also leads to neuroinflammatory responses, including, ADAM10, and ADAM17 activation, and causes the secretion of prostaglandin E2 and proinflammatory cytokines, such as interleukin-1β (IL-1β), IL-2, IL-4, IL-6, and tumor necrosis factor (TNF) (Engel et al., 2012b; Beamer et al., 2017). P2X7Rs mediate NLRP3 inflammasome-dependent IL-1β secretion following activation of NF-κB in the brain and immune cells (Albalawi et al., 2017; Burnstock and Knight, 2018). Therefore, P2X7Rs are a target for neurodegenerative diseases.

P2X7R expression has been widely shown in the central nervous system, including microglia, oligodendrocytes, and ependymal cells (Jimenez-Mateos et al., 2019). The P2X7R is mainly expressed on microglia, but not on the neurons (Jimenez-Pacheco et al., 2016; Kaczmarek-Hajek et al., 2018). P2X7R is expressed in the CA1 pyramidal and dentate granule neurons, as well as in microglia of epileptic mice (Jimenez-Pacheco et al., 2016) and in the neurons of rat hippocampus (Sperlagh et al., 2002). Moreover, P2X7R expression has been detected in both the astrocyte culture of brain/spinal cord slices (Illes et al., 2012, 2017). This conflicting data about functional expression of P2X7R could be attributed to both brain region-specific expression and the pathological conditions of the brain such as epilepsy (Beamer et al., 2017). P2X7R expression has increased in the hippocampus and neocortex regions of the brain in many epilepsy models (Jimenez-Pacheco et al., 2016; Huang et al., 2017; Rodriguez-Alvarez et al., 2017; Zeng et al., 2017; Jimenez-Mateos et al., 2019).

Absence epilepsy is a common neurological disease in children that affects educational success. Absence epilepsy is a loss of consciousness with a sudden pause in behavior, and if electroencephalography is recorded during seizures, bilateral synchronous 3-Hz frequency spike-wave discharges (SWDs) are observed (Russo et al., 2016; Fisher et al., 2017). Its pathophysiology is unclear, but it has been shown that absence seizures start from a glutamatergic focus located in the perioral region of the somatosensory cortex, and then this area affects the thalamus over time and creates a cortico-thalamo-cortical circuit (Meeren et al., 2005; Pinault and O’Brien, 2005). Wistar Albino Glaxo/Rijswijk (WAG/Rij) rats are also a validated genetic model of absence epilepsy characterized by SWDs on electroencephalography with a spontaneous pause in behavior (Russo et al., 2016). IL-1β increases in the somatosensory cortex and IL-1β antagonist administration reduces SWDs in the Genetic Absence Epilepsy Rat from Strasbourg (GAERS) model, which is also a validated genetic absence epilepsy model (Akin et al., 2011).

The effects of P2X7Rs on epilepsy have been investigated in various experimental epilepsy models (Fischer et al., 2016; Huang et al., 2017; Nieoczym et al., 2017; Rodriguez-Alvarez et al., 2017; Arslan et al., 2019). In a kainic acid-induced status epilepticus (SE) model, P2X7R expression was shown to increase, and seizure severity and neuronal death decreased after pre-treatment or post-treatment with intracerebroventricular (i.c.v.) injection of A-438079 or systemic administration of JNJ-47965567 (Engel et al., 2012a; Jimenez-Pacheco et al., 2013, 2016). Systemic administration of A-438079 reduced convulsions in the kainic acid-induced SE model and reduced neuronal death more than phenobarbital in 10-day-old rats (Mesuret et al., 2014). In a penicillin-induced epilepsy model, a P2X7R agonist showed proconvulsant effects that could be reversed by A-438079 and a T-type calcium channel blocker, whereas a P2X7R antagonist, A-438079, showed an anticonvulsant effect (Arslan et al., 2019). In a pentylenetetrazol (PTZ) kindling model, P2X7R antagonists, Brilliant Blue G (BBG) and tanshinone showed a slight delay in kindling development, and JNJ-47965567 and AFC-5128 showed a strong delay (Soni et al., 2015; Fischer et al., 2016). However, P2X7R antagonists were ineffective in fully kindled rats (Fischer et al., 2016). They also did not affect the number of seizures in the kainic acid-induced kindling model, but they gave rise to less severe chronic seizures (Amhaoul et al., 2016). Systemic administration of A-438079 reduced acute seizures during hypoxia in neonatal mice but had no effect on post-hypoxia seizures (Rodriguez-Alvarez et al., 2017). P2X7R antagonists did not affect the maximal electroshock seizure threshold test or PTZ seizure threshold test, but AFC-5128 or JNJ-47965567 combination with carbamazepine increased the seizure threshold more than carbamazepine alone (Fischer et al., 2016; Nieoczym et al., 2017). BBG showed a week anticonvulsant action on the threshold of 6 Hz induced psychomotor seizures in mice (Nieoczym et al., 2017). However, P2X7R knockout mice were more susceptible to pilocarpine-induced seizures (Kim and Kang, 2011), and blockade of P2X7Rs increased the number and severity of pilocarpine-induced seizures in mice (Rozmer et al., 2017).

On the other hand, NMDA receptors are cation channels, and over-stimulation leads to an increase in intracellular calcium, which could be toxic for cells (Rosini et al., 2019). Memantine, a non-competitive NMDA receptor antagonist, has also shown anticonvulsant effects in many experimental epilepsy models, including a penicillin-induced epilepsy model, an audiogenic seizure model, and a PTZ kindling model (Frey and Voits, 1991; Cakil et al., 2011; Kim et al., 2012; Zaitsev et al., 2015). Only one study has been conducted with WAG/Ola/Hsd rats, which are thought to be a model of genetic absence epilepsy (Frey and Voits, 1991), but there is no much information about this rat substrain. The impairment of gamma-aminobutyric acid (GABA) and glutamate leads to epilepsy. GABAergic antiepileptic drugs worsen absence seizures (Panayiotopoulos, 2001). However, the NMDA receptor NR1 subunit showed a decrease depending on age in WAG/Rij rats (van de Bovenkamp-Janssen et al., 2006). In addition, in both 2-month-old and 6-month-old WAG/Rij rats, the NR2B subunit was lower in various layers of the somatosensory cortex than in the Wistar rats of the same age (Karimzadeh et al., 2013). Stimulation of NMDA receptors increased SWDs in WAG/Rij rats, whereas NMDA receptor reduced SWDs (Peeters et al., 1994).

The P2X7R increases glutamate secretion in a vesicular and non-vesicular manner (Sperlagh et al., 2002; Cho et al., 2010). It also affects GABA and glutamate reuptake in a calcium-dependent manner (Barros-Barbosa et al., 2018). The P2X7R was found to be non-desensitizing, and it allows substantial calcium influx (Fischer et al., 2016). It activates intracellular signaling pathways (Takenouchi et al., 2010). Both P2X7R and NMDA receptor activation can activate pathways of reactive oxygen species (ROS) (Davidson and Duchen, 2006). ROS cause many changes such as aging, cardiovascular disease, cancer, and neurodegenerative diseases, including epilepsy, by damaging proteins, lipids, carbohydrates, and nucleic acids, and they can be controlled by antioxidant systems, preventing the formation of ROS, and damage (Droge, 2002; Terrone et al., 2019).

x There are limited data on the interaction between P2X7Rs and NMDA receptors in epilepsy. Thus, the effects of P2X7Rs and NMDA receptors and their relationship were investigated using a P2X7R agonist, BzATP, which is more selective for P2X7R than for other P2X receptors, a P2X7R antagonist, A-438079, which highly targets the P2X7R, and a selective antagonist of the NMDA receptor, memantine, in WAG/Rij rats with both electrophysiological and biochemical analysis methods.

## Materials and Methods

### Animals

In this study, 63 male, 6–8 months old, 250–300 g weighing WAG/Rij rats were used with the permission of Ondokuz Mayis University Animal Experiments Local Ethics Committee (2015/56). Wistar Albino Glaxo/Rijswijk (WAG/Rij) rats with spontaneous seizures were purchased from Charles River Lab (Germany). Animals were fed *ad libitum* and housed in heat-regulated rooms, on a 12 h light-dark cycle. All experimental procedures were conducted under the European Union Directive (2010/63/EU), and Turkish legislation acts concerning animal experiments.

Animals were divided into nine groups (*n* = 7) randomly as follows:

1. Control group (WAG/Rij rat)
2. Sham Group (WAG/Rij rat, 2 μl sterile distilled water; i.c.v.)
3. BzATP 50 μg (i.c.v.) group
4. BzATP 100 μg (i.c.v.) group
5. A-438079 20 μg (i.c.v.) group
6. A-438079 40 μg (i.c.v.) group
7. Memantine 5 mg/kg (i.p.) group
8. BzATP 100 μg (i.c.v.) + Memantine 5 mg/kg (i.p.) group
9. A-438079 20 μg; (i.c.v.) + Memantine 5 mg/kg (i.p.) group.

### Surgery and Electrocorticography Recording

Animals were anesthetized and sedated with ketamine/xylazine (90/10 mg/kg; i.p.) and placed in the stereotaxic apparatus. After the skin was cut off about 3 cm and folded back, subcutaneous tissue was removed from the cranium. According to the rat brain atlas (Paxinos and Watson, 2007), four burr holes were drilled in the skull with a microdrill without damaging the dura mater. For ECoG recordings, three screw electrodes were placed into the holes as coordinates: first electrode; 2 mm anterior and 3.5 mm right lateral to bregma, second electrode; 6 mm posterior and 4 mm right lateral to bregma and earth electrode was placed on the cerebellum. For i.c.v. injections, an external cannula was advanced into the brain as coordinates: 1.1 mm posterior, 1.5 mm right lateral and 3.2 mm vertical to bregma. Afterward, the electrodes and cannula were fixed to the skull with dental cement. The cannula was covered with a dust cup until used. After the surgery, animals were housed individually for a week and habituated to the recording cage (32 cm × 30 cm in width, 50 cm high) for 3 days before the experimental procedure.

On the experiment day, animals were connected to the ECoG recording system (PowerLab, 16/SP, AD Instruments, Australia) by an isolated flexible cable. Baseline electrocorticography (ECoG) recordings were taken for 3 h from all awake animals at the same time of day (10:00 AM). After the drug injection, ECoGs recording continued for another 3 h (Aygun et al., 2019). The number of SWDs and the mean duration and amplitude of SWDs were measured and calculated for every 20 min offline with LabChart 7 Pro (AD Instruments, Australia) (Arslan et al., 2013, 2014).

### Drugs and Drug Administration

Ketamine hydrochloride, xylazine hydrochloride, A-438079 hydrochloride hydrate, BzATP [2′(3′)-*O*-(4-Benzoylbenzoyl) adenosine 5′-triphosphate triethylammonium] and memantine hydrochloride were purchased from Sigma Chemical Co., St. Louis, MO, United States and dissolved in sterile distilled water. After obtaining 180 min of baseline ECoG recordings, A-438079, at the doses of 20 and 40 μg, and BzATP, at the doses of 50 and 100 μg were administered into the lateral ventricle within a thin internal cannula (4.2 mm vertical to the bregma) in a volume of 2 μl. Memantine, at a dose of 5 mg/kg, was injected intraperitoneally in a volume of 0.5 ml. For the interaction groups, memantine was administered 10 min after the chosen doses of BzATP (100 μg) or A-438079 (20 μg) (Arslan et al., 2019). The sham group was given sterile distilled water (2 μl, i.c.v.).

### Biochemical Analysis

After the end of the ECoG recordings, all rats were decapitated following ketamine/xylazine anesthesia. Right and left hemisphere, cerebellum, and brainstem were separated in oxygenated artificial cerebrospinal fluid [aCSF containing (mM): NaCl, 124; KCl, 5; KH2PO4, 1.2; CaCl_2_, 2.4; MgSO_4_, 1.3; NaHCO_3_, 26; glucose, 10; HEPES, 10, at pH 7.4 when saturated with 95% O_2_ and 5% CO_2_]. The tissues were frozen in liquid nitrogen, homogenized immediately, and soaked in phosphate buffer solution (PBS, 10 mM, and pH 7.2). After 1 min of sonication at +4°C with 220 V (METU Electromechanical, Germany), homogenates were stored at −80°C. On the evaluation day, the homogenates were defrosted at room temperature and were centrifuged at +4°C for 5 min with 15,000 × *g* (Sigma 3K30, Germany) for biochemical analysis.

Tissue protein concentrations were determined with Lowry’s method (Lowry et al., 1951). The results were expressed per mg protein. Advanced protein oxidation products (AOPP), malondialdehyde (MDA), superoxide dismutase (SOD), catalase (CAT), glutathione (GSH), glutathione peroxidase (GPx) and glutathione reductase (GR) concentrations in the tissues were examined with commercial rat ELISA kits (SunRed Biotechnology Co., Shanghai, China). These kits all use a double-antibody sandwich enzyme-linked immunosorbent assay (Arslan et al., 2019).

### Statistical Analysis

Spike-wave discharges parameters were calculated, by using the raw data obtained from LabChart 7-Pro, with an excel program. The total number of SWDs after drug injections was calculated for every 20 min and these data were converted to percentage values by comparing to the baseline values. The mean duration and the amplitude of SWDs during 180 min after injections were calculated as a percentage by comparing to the baseline data.

SPSS 15.0 data analysis software was used for statistical analyses. The normality of the data was tested with the Shapiro–Wilk test. After verifying that the data were normally distributed paired-samples *t*-test was performed between dependent groups, and one- or two-way ANOVA and then *post hoc* Tukey tests were used for multiple comparisons. The results are expressed as mean ± standard error (SEM). For all statistical analyses, *p* < 0.05 was considered statistically significant.

## Results

**FIGURE 1:**
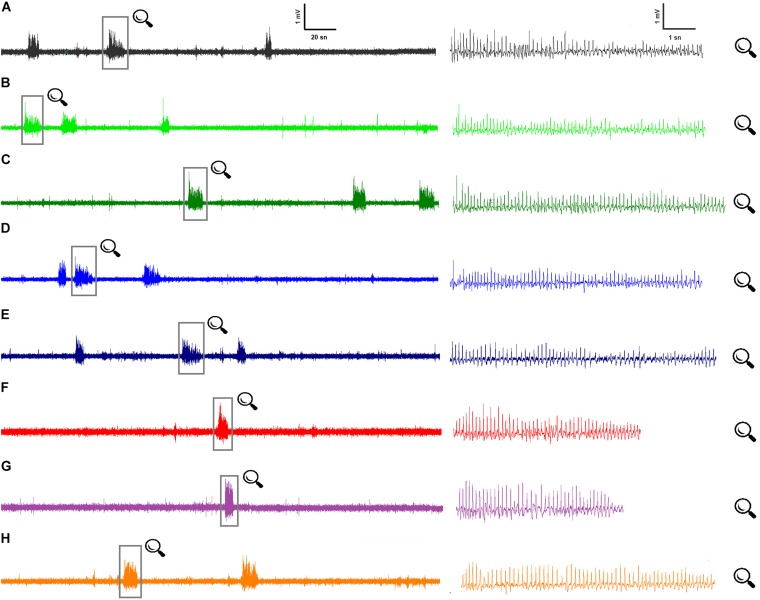
Representative ECoG recordings for all groups at 80th minute: **(A)** Control ECoG activity in WAG/Rij rats (*n* = 7); **(B)** BzATP, at the dose of 50 μg, i.c.v. (*n* = 7); **(C)** BzATP, at dose of 100 μg, i.c.v. (*n* = 7); **(D)** A-438079, at dose of 20 μg, i.c.v. (*n* = 7); **(E)** A-438079, at dose of 40 μg, i.c.v. (*n* = 7); **(F)** Memantine administration, at dose of 5 mg/kg, i.p. (*n* = 7); **(G)** BzATP (100 μg) administration 10 min before memantine (*n* = 7); **(H)** A-438079 (20 μg) administration 10 min before memantine (*n* = 7).

All rats showed SWDs in ECoG characterized by paroxysmal unresponsiveness to environmental stimuli (Figure 1). Paired-samples *t*-test revealed that the total numbers and the mean duration of SWDs did not significantly change after the injection of solvent compared to baseline values. The total numbers and mean durations of SWDs were 108.4 ± 4.8, 111.0 ± 3.8 and 8.67 ± 0.18, 8.76 ± 0.21 s during 180 min before and after the administration of sterile distilled water, respectively. The mean amplitudes of SWDs were 0.635 ± 0.039 and 0.648 ± 0.048 mV before and after the administration of sterile distilled water, respectively. There was no statistical difference regarding the parameters of SWDs between the control and sham groups (*p* > 0.05).

Table 1 shows the biochemical analysis for the left and right hemispheres, cerebellum, and brainstem of the groups. There was no significant difference between the sham group and the control group in any of the biochemical parameters.

**TABLE 1:** The levels of advanced oxidation protein products (AOPP), malondialdehyde (MDA), superoxide dismutase (SOD), glutathione (GSH), catalase (CAT), glutathione peroxide (GPx), and glutathione reductase (GR) in the left and right hemispheres, cerebellum and brainstem of all experimental groups.

		**AOPP**	**MDA**	**SOD**	**GSH**	**CAT**	**GPx**	**GR**
		**(nmol/mL)**	**(nmol/mL)**	**(ng/mL)**	**(ng/mL)**	**(ng/mL)**	**(ng/mL)**	**(ng/mL)**
LEFT HEMISPHERE	Control	4.7 ± 0.7	5.7 ± 0.8	5.1 ± 0.5	52.8 ± 5.6	10.8 ± 0.8	8.8 ± 0.7	5.9 ± 0.5
	BzATP (100 μg)	5.65 ± 0.9	6.7 ± 0.8	7.1 ± 0.6*	60.6 ± 5.5	10.1 ± 0.7	9.7 ± 1.04	8.7 ± 1.1*
	A-438079 (20 μg)	4.4 ± 0.5	6.04 ± 0.7	4.6 ± 0.2	48.4 ± 4.3	11.8 ± 1.3	6.4 ± 0.9	4.7 ± 0.7
	Memantine (5 mg/kg)	3.6 ± 0.5	2.6 ± 0.4*	4.6 ± 0.4	35.02 ± 3.5*	17.3 ± 0.6**	7.6 ± 0.6	2.6 ± 0.3**
	BzATP + memantine	4.9 ± 0.7	4.7 ± 0.6	4.5 ± 0.7	37.4 ± 2.9*	15.06 ± 0.3*	9.5 ± 1.1	3.7 ± 0.3*
	A-438079 + memantine	3.4 ± 0.6	3.1 ± 0.3*	4.3 ± 0.6	32.2 ± 4.1*	16.1 ± 0.5*	6.9 ± 0.4	3.07 ± 0.4*
RIGHT HEMISPHERE	Control	5.02 ± 1.03	5.8 ± 0.7	5.06 ± 0.4	47.5 ± 4.7	11.3 ± 1.8	7.6 ± 0.5	5.7 ± 0.3
	BzATP (100 μg)	6.1 ± 1.07	7.1 ± 1.1	8.03 ± 0.8*	56.4 ± 53	10.5 ± 1.3	8.8 ± 0.8	8.1 ± 0.5*
	A-438079 (20 μg)	4.6 ± 0.7	5.06 ± 1.01	4.5 ± 0.3	50.6 ± 5.1	9.9 ± 2.01	7.04 ± 0.33	5.07 ± 0.4
	Memantine (5 mg/kg)	4.5 ± 0.6	3.1 ± 0.4*	5.2 ± 0.6	27.9 ± 3.1*	18.2 ± 0.7*	6.04 ± 0.7	2.5 ± 0.4**
	BzATP + memantine	6.09 ± 0.9	4.7 ± 0.6	5.2 ± 0.7	25.5 ± 3.09*	19.7 ± 1.3*	6.4 ± 0.8	3.4 ± 0.5*
	A-438079 + memantine	4.2 ± 1.02	3.3 ± 0.3*	4.6 ± 0.4	29.06 ± 4.1*	17.8 ± 0.6*	5.7 ± 0.4	2.4 ± 0.6**
CEREBELLUM	Control	11.2 ± 1.3	7.4 ± 1.05	9.07 ± 1.06	73.8 ± 8.3	15.7 ± 1.9	10.5 ± 1.3	9.1 ± 0.5
	BzATP (100 μg)	12.1 ± 1.2	7.6 ± 1.07	10.3 ± 0.8	78.5 ± 2.06	15.6 ± 2.4	12.5 ± 0.9	9.02 ± 0.4
	A-438079 (20 μg)	10.3 ± 1.01	6.6 ± 0.9	8.4 ± 0.9	62.8 ± 8.4	13.7 ± 2.1	10.7 ± 1.05	8.4 ± 0.8
	Memantine (5 mg/kg)	8.9 ± 0.7	4.4 ± 0.6*	7.7 ± 1.2	60.02 ± 7.1	20.7 ± 2.4	9.7 ± 0.7	7.9 ± 0.8
	BzATP + memantine	9.1 ± 1.1	7.08 ± 0.8	7.2 ± 1.1	66.4 ± 6.5	17.6 ± 3.4	8.7 ± 1.05	10.03 ± 1.2
	A-438079 + memantine	7.7 ± 1.4	3.6 ± 0.5*	8.03 ± 0.8	59.7 ± 5.9	19.9 ± 3.6	8.6 ± 0.8	8.9 ± 0.7
BRAINSTEM	Control	9.3 ± 0.9	11.8 ± 1.2	7.1 ± 0.5	100.4 ± 9.9	18.1 ± 1.9	17.8 ± 2.6	8.05 ± 1.1
	BzATP (100 μg)	10.1 ± 1.3	13.3 ± 0.9	9.3 ± 0.7	103.7 ± 11.7	17.9 ± 1.7	19.7 ± 2.1	8.4 ± 0.6
	A-438079 (20 μg)	8.6 ± 0.9	10.7 ± 0.6	6.8 ± 0.6	108.4 ± 11.05	19.5 ± 3.08	17.45 ± 1.3	7.1 ± 0.7
	Memantine (5 mg/kg)	7.6 ± 0.7	6.4 ± 1.1*	6.3 ± 0.8	92.3 ± 10.5	17.8 ± 2.1	16.9 ± 1.4	7.3 ± 0.8
	BzATP + memantine	8.3 ± 0.8	9.1 ± 0.8	5.9 ± 0.4	89.7 ± 8.8	21.8 ± 3.4	14.3 ± 1.2	10.1 ± 1.2
	A-438079 + memantine	7.5 ± 1.05	7.1 ± 0.7**	6.09 ± 0.6	82.3 ± 7.1	14.6 ± 1.7*	16.5 ± 1.07	7.6 ± 0.7

*p < 0.05, **p < 0.01 compared to control group.

### The Role of P2X7Rs in WAG/Rij Rats

**FIGURE 2:**
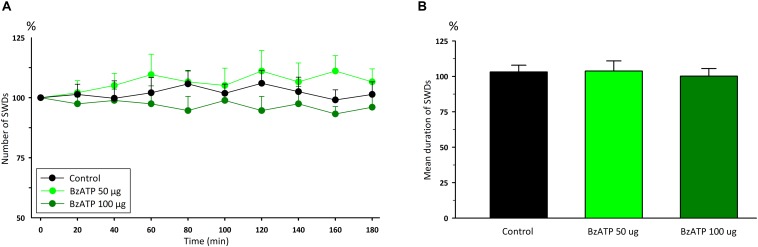
The effects of P2X7R agonist BzATP on **(A)** the total number of SWDs in every 20 min and **(B)** the mean duration of SWDs for 180 min.

**FIGURE 3:**
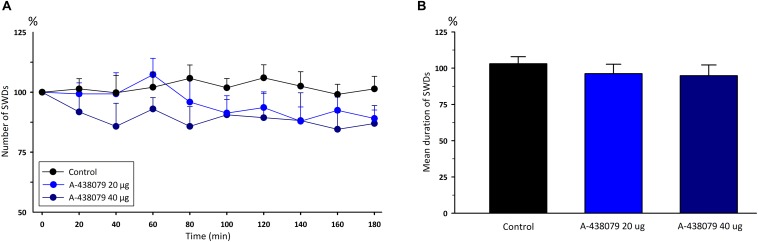
The effects of P2X7R antagonist A-438079 on **(A)** the total number of SWDs in every 20 min and **(B)** the mean duration of SWDs for 180 min.

Administration of P2X7Rs agonist BzATP, at the doses of 50 and 100 μg, did not significantly change any of the total numbers [*F*_(8,162)_ = 0.12] and durations [*F*_(2,18)_ = 1.54] of SWDs compared to the control group (Figure 2). Total numbers, mean durations and mean amplitudes of SWDs were 12.2 ± 0.9, 11.1 ± 0.9; 9 ± 0.4, 8.9 ± 0.4 s;0.6490 ± 0.013, 0.6148 ± 0.016 mV in the 80th minute in the BzATP 50 μg and BzATP 100 μg groups, respectively. After the injections of BzATP, at doses of 50 and 100 μg, the total numbers and mean durations of SWDs were 107.3 ± 4.2, 102.7 ± 5.4; 8.82 ± 0.38, 8.52 ± 0.29 s during 180 min, respectively. Injection of A-438079, at the doses of 20 and 40 μg, did not alter the total numbers [*F*_(8,162)_ = 0.54] and durations [*F*_(2,18)_ = 6.54] of SWDs compared to the control group (Figure 3). The total numbers and mean durations of SWDs were 107.8 ± 7.7, 102.7 ± 9.4; 8.38 ± 0.28, 8.2 6 ± 0.34 s during 180 min after the injections of 20 and 40 μg A-438079, respectively.

BzATP, at a dose of 100 μg, injection significantly increased SOD and GR levels in the left and right hemispheres (*p* < 0.05). Other biochemical parameters were not different in the BzATP group compared to the control group (*p* > 0.05). A-438079, at a dose of 20 μg, did not alter any of the biochemical parameters (Table 1).

### The Effect of Memantine on WAG/Rij Rats

**FIGURE 4:**
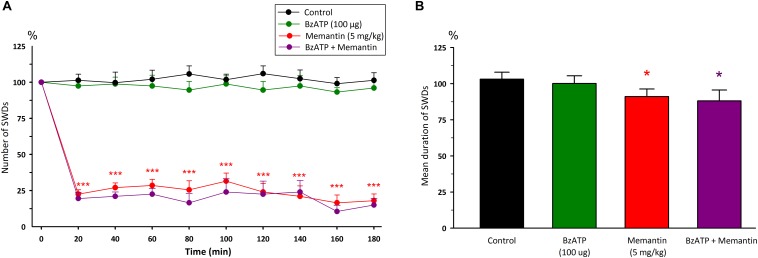
The effects of memantine at a dose of 5mg/kg and its interaction with BzATP on **(A)** the total number of SWDs in every 20 min and **(B)** the mean duration of SWDs for 180 min. *p < 0.05, ***p < 0.001 compared to control group.

Intraperitoneal injection of memantine (5 mg/kg) significantly decreased the total number [*F*_(8,108)_ = 0.66, *p* < 0.001] and mean duration of SWDs [*F*_(1,12)_ = 6.34, *p* < 0.05] 20 min after injection compared to control group (Figure 4). The total number, mean duration and amplitude of SWDs were 32.5 ± 3.6; 7.74 ± 0.19 s and 0.635 ± 0.03 mV during 180 min after the administration of memantine, respectively.

Memantine significantly decreased MDA levels in all brain regions compared to the control group (*p* < 0.05). In the cerebrum, injection of memantine decreased GSH and GR levels, and increased CAT levels (*p* < 0.05). Other biochemical parameters were not different in the memantine group compared to the control group (Table 1).

### Interaction Between P2X7R and Memantine in WAG/Rij Rats

Injection of BzATP (100 μg) 10 min before memantine decreased the total number of SWDs after 20 min [*F*_(8,216)_ = 1.10, *p* < 0.001] and the mean duration of SWDs [*F*_(3,24)_ = 10.60, *p* < 0.05] compared to the control group (Figure 4). However, the total number and the mean duration of SWDs were not found to be different after the co-administration of BzATP with memantine compared to the alone memantine injection. The total number and the mean duration of SWDs were 26.3 ± 2.8 and 7.56 ± 0.42 s for 180 min after the co-administration of BzATP with memantine, respectively.

Co-administration of BzATP with memantine decreased the levels of GSH and GR, and increased CAT levels in the cerebrum compared to the control group (*p* < 0.05). Other biochemical parameters did not alter with the co-administration of BzATP with memantine compared to the control group.

**FIGURE 5:**
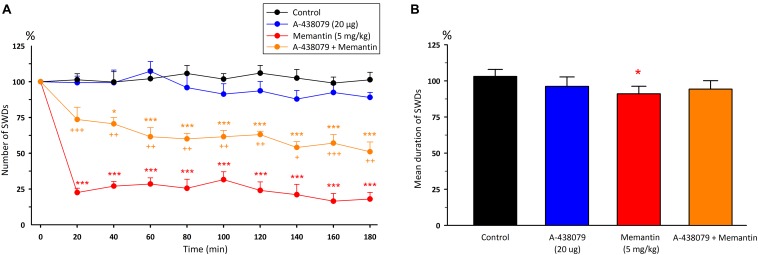
The effects of memantine at a dose of 5mg/kg and its interaction with A-438079 on **(A)** the total number of SWDs in every 20 min and **(B)** the mean duration of SWDs for 180 min. *p < 0.05, ***p < 0.001 compared to the control group. ^+^*p* < 0.05, ^++^*p* < 0.01, ^+++^*p* < 0.001 compared to the – memantine group.

Although administration of A-438079 10 min prior to memantine decreased the total number of SWDs after 40 min compared to the control group (*p* < 0.05), but it appears that A-438079 partially reversed the anticonvulsant activity of memantine after 20th minute compared to the memantine group alone [*F*_(8,216)_ = 1.97, *p* < 0.001]. The mean duration of SWDs did not alter compared to both the control and memantine groups [*F*_(3,24)_ = 6.74, Figure 5]. The total number and mean duration of SWDs were 59.7 ± 6.6 and 8.05 ± 0.21 s for 180 min after the co-administration of A-438079 with memantine, respectively.

Malondialdehyde levels significantly decreased in all regions after the co-administration of A-438079 with memantine (*p* < 0.05). In the cerebrum, the combination of A-438079 + memantine decreased GSH and GR levels, and increased CAT levels compared to the control group (*p* < 0.05). Interestingly, CAT levels were decreased in the brainstem with the injections of A-438079 and memantine (*p* < 0.05). The rest of the measured biochemical parameters were not changed in the A-438079 + memantine group compared to the control group (Table 1).

## Discussion

In the present study, the electrophysiological results revealed that the administration of memantine showed an anticonvulsant effect without changing the mean amplitude of SWDs. Neither BzATP nor A-438079 affected the parameters of SWDs in WAG/Rij rats. BzATP did not reverse the anticonvulsant activity of memantine. However, A-438079 reduced the anticonvulsant activity of memantine.

BzATP increased only the SOD and GSH levels in the cerebrum, whereas A-438079 did not significantly affect any of the biochemical parameters. Memantine showed antioxidant effects by decreasing MDA levels in all tissue samples. Memantine also reduced GSH and GR and increased CAT levels in the cerebrum. BzATP reversed the antioxidant effects of memantine on MDA, while A-438079 enhanced the anticonvulsant effects of memantine in the brainstem.

### The Role of P2X7R in WAG/Rij Rats

The P2X7R is the newest member of the purinergic receptor family and has been the subject of epilepsy research with its widespread presence in the nervous system (Jimenez-Mateos et al., 2019). The involvement of the P2X7R in epilepsy has been demonstrated by several researchers with either anticonvulsant, proconvulsant, or no effects in various models of epilepsy (Jimenez-Pacheco et al., 2016; Rodriguez-Alvarez et al., 2017; Arslan et al., 2019). These controversial results depend on experimental differences, such as the selection of the epilepsy model, the dosage of the P2X7R agonist and antagonist used, and the species of the experimental animals. We used an absence epilepsy model in this study, which was a chronic non-convulsive epilepsy model, and there are some unique mechanisms known to create absence seizures (Meeren et al., 2005; D’Antuono et al., 2006; Luttjohann and van Luijtelaar, 2012; Scicchitano et al., 2015). The P2X7R is a non-selective cation channel, and it allows calcium influx, which is essential for the absence epilepsy pathogenesis.

BzATP and A-438079 have high potency for the P2X7R. BzATP is the most potent for the P2X7R, but it is not a selective P2X7R agonist (Donnelly-Roberts and Jarvis, 2007). Thus, BzATP may also be effective in other P2X receptors that may also have an important role in epilepsy (Michel et al., 2001; Henshall et al., 2013). The potent inhibition of the P2X7R with A-438079 confirms that the P2X7R accounts for ATP-triggered Ca^2+^ entry (Fischer et al., 2016). The moderate bioavailability and moderate plasma elimination half-life were 19% and 1.02 h, respectively, for intraperitoneal A-438079 in rats (McGaraughty et al., 2007). Brain levels of A-438079 rapidly declined after parenteral injection, and Mesuret et al. (2014) suggested that the rapid elimination of the compound restricts the therapeutic window of this compound. However, in our previous study, i.c.v. injection of BzATP and A-438079 showed their effects on the penicillin-induced epileptiform activity within 20 and 60 min after their injections, respectively, and lasted for 120 min (Arslan et al., 2019). BzATP is the most potent agent for the P2X7R, and A-438079 is specific and can almost abolish the effects of BzATP (Anderson and Nedergaard, 2006; Fischer et al., 2016). The multi-targeting of different P2X receptors and their stability in the brain may be considered possible limitations for such studies in epilepsy. In addition, P2X7Rs release cytokines during normal brain function (Sperlagh and Illes, 2014). In particular, P2X7R activation might be linked to the regulation of various aspects of immunocompetent cells through the expression and secretion of many inflammatory mediators, including IL-1b, IL-2, IL-4, IL-6, IL-8, and TNFα (Engel et al., 2012b). However, inflammatory mediators were not measured, which might be another limitation of the present study. Therefore, further studies are required to determine both receptor-specific localization and the level of inflammatory mediators in an absence epilepsy model.

In an Alzheimer’s disease model, P2X7R activity affected nicotinamide adenine dinucleotide phosphate (NADPH) activity and increased the formation of the O^.–^ radical by acting on p38 MAPK (Parvathenani et al., 2003). The antioxidant enzyme that converts the O^.–^ radical into H_2_O_2_ is SOD, and an increase in the SOD level promotes the increase of O^.–^ radicals (Younus, 2018). Activation of the P2X7R due to BzATP promotes ROS production through NADPH oxidase in macrophages, microglia, and neurons that can be blocked by P2X7R inhibitors (Hewinson and Mackenzie, 2007; Mead et al., 2012; Munoz et al., 2017). The P2X7R has also been shown to directly affect SOD in amyotrophic lateral sclerosis models and cell culture studies (Gandelman et al., 2013; Fabbrizio et al., 2017). GR is used in the reduction reaction of NADPH to GSH disulfide, which is a way to decrease NADPH. BzATP increases the production and release of ROS in the substantia gelatinosa of the spinal cord by stimulating P2X7Rs in astroglia (Ficker et al., 2014). However, Safiulina et al. (2006) showed ATP-induced ROS generation in CA3 pyramidal neurons due to the stimulation of P2Y1 receptors, not P2X7Rs. ATP treatment increased the expression of Cu/Zn SOD in the RBA-2 astrocyte cell line of cell culture (Chen et al., 2006). In our previous study, BzATP increased lipid peroxidation and the levels of protein oxidation and antioxidant proteins in the brain of penicillin-induced epileptic rats (Arslan et al., 2019). In the present study, BzATP and A-438079 did not affect protein or lipid oxidation, but BzATP increased SOD and GR in the cerebrum. Accordingly, the P2X7R seems to be more effective in the cerebral cortex in WAG/Rij rats with absence epilepsy. Since it has been suggested that oxidative mechanisms and inflammatory mechanisms do not cause the formation of absence epilepsy (Grosso et al., 2011) and there are no inflammatory processes in WAG/Rij rats, it is logical to expect that the low number of P2X7Rs and efficacy may be the reason for the ineffectiveness of BzATP and A-438079 observed in the present study in absence epileptic rats. However, children with absence epilepsy have shown no oxidant markers, while increased lipid peroxidation and protein oxidation levels have been observed in epileptic encephalopathic patients (Grosso et al., 2011).

### The Role of NMDA Receptors in WAG/Rij Rats

Although memantine is mostly used in Alzheimer’s disease to improve cognitive function, the anticonvulsant activity of memantine has been demonstrated in various models of experimental epilepsy (Frey and Voits, 1991; Cakil et al., 2011; Kim et al., 2012; Zaitsev et al., 2015). Memantine showed an anticonvulsant effect both in a penicillin-induced epilepsy model and in Krushinsky–Molodkina rats with audiogenic seizures (Cakil et al., 2011; Kim et al., 2012). Memantine was also effective on the tonic component of seizures in a PTZ kindling model, and it prevented neuronal death (Zaitsev et al., 2015). Moreover, memantine showed anticonvulsant effects in WAG/Ola/Hsd rats, which are thought to be a model of genetic absence epilepsy (Frey and Voits, 1991), but there is no sufficient information about this rat substrain. In agreement with these studies, memantine has an anticonvulsant effect on absence epilepsy in WAG/Rij rats, which was used in the present study. Recent studies have shown that the initial focal point of absence epilepsy is in the perioral area of the somatosensory cortex and that the cortex is present in NMDA receptor-mediated glutamatergic pyramidal neurons and is present in layers V and VI (Miras-Portugal et al., 2003; Scicchitano et al., 2015; Russo et al., 2016). Thus, it can be concluded that seizures can be prevented without initially entering the thalamo-cortical circuit by decreasing the activity in this region in the presence of memantine.

As an NMDA receptor blocker, memantine has an antioxidant effect by blocking calcium entry, affecting intracellular signaling pathways (Flores et al., 2011; Annweiler and Beauchet, 2012; Gubandru et al., 2013; Lee et al., 2018). Memantine decreased the level of MDA, which is the final product of polyunsaturated fatty acid peroxidation, in all brain tissue samples and decreased GSH and GR levels but increased CAT levels in the cerebrum in this study.

### Functional Interaction Between P2X7Rs and NMDA Receptors

Pre-treatment of organotypic hippocampal slice cultures with ATP reduced NMDA-induced neuronal death in microglia, suggesting microglia-mediated neuroprotection depends on P2X7Rs (Masuch et al., 2016). The glutamatergic agonists NMDA and AMPA increased the BzATP-induced current amplitudes in organotypic hippocampal slice cultures (Khan et al., 2018). Intravitreal injection of A-438079 and BBG significantly reduced NMDA-induced cell loss in the retinae of male Wistar rats, suggesting a strong link between P2X7R and NMDA (Sakamoto et al., 2015). Activation of the P2X7R has been shown to trigger the release of glutamate from neurons and astrocytic cells by vesicular and non-vesicular pathways (Sperlagh et al., 2002; Cho et al., 2010). Stimulation of NMDA receptors also increases ATP release (Cho et al., 2010; Engel et al., 2012a, b). In addition, P2X7Rs have been observed on presynaptic in glutamatergic pyramidal neurons (Sperlagh et al., 2002; Miras-Portugal et al., 2003), suggesting a P2X7-NMDA receptors interaction in pyramidal cells located in the perioral region of the somatosensory cortex, which is considered to be the initial focal point of absence epilepsy. In addition, both receptors are known to increase the intracellular calcium level. Thus, the interaction between P2X7 and NMDA receptors may increase excitability in the brain and may cause neurotoxicity by increasing ROS (Carrasco et al., 2018). In a phencyclidine-induced schizophrenia model, prefrontocortical postsynaptic NMDA currents slightly decreased due to both genetic deletion (P2X7R -/-) and pharmacological blockade with JNJ-47965567 (Kovanyi et al., 2016). However, NMDA currents were not affected in either wild type or P2X7R-deficient mice in *in situ* cortical astroglia (Oliveira et al., 2011). In contrast, electrophysiological recordings revealed that stimulation of the P2X7R with BzATP did not reverse memantine’s anticonvulsant effect; the anticonvulsant activity of memantine was maintained in the presence of BzATP, but it neutralized the level of MDA. The administration of A-438079 reduced the anticonvulsant activity of memantine, but the net effect remained anticonvulsive in this study. This finding might have been due to increased glutamate through BzATP activity, leading to cytotoxic effects through other glutamate receptors, such as AMPA and kainate (Coyle and Puttfarcken, 1993; Quincozes-Santos et al., 2014). There is evidence that some intracellular pathways affect the interaction of the two receptors. They both affect phosphokinase C (Ortega et al., 2010), pannexin-1 (Bravo et al., 2015), and the MAP kinase pathway (Gandelman et al., 2013; Fabbrizio et al., 2017; Lee et al., 2018). Therefore, it appears that this effect is due to the possible interaction of P2X7 and NMDA receptors in intracellular pathways. In addition, A-438079 and memantine decreased MDA levels in all tissues and changed antioxidant parameters similar to the memantine group in the present study, suggesting a strong interaction between the P2X7R and NMDA receptors in the brainstem.

## Conclusion

Electrophysiological data from the present study suggest that P2X7Rs are ineffective for absence epilepsy whereas a biochemical analysis revealed a partial interaction between P2X7 and NMDA receptors in WAG/Rij rats with absence epilepsy. It seems logical to expect this interaction since P2X7R and the NMDA receptor both allow calcium influx. P2X7 and NMDA receptors use common intracellular signal pathways, but this interaction cannot be limited to calcium influx in epilepsy. Many other systems and receptors are involved in calcium influx without P2X7 or NMDA receptors and contribute to epileptogenesis. Besides, the P2X7R has been linked to inflammatory mediators in neurological diseases. Therefore, further studies are required to determine the level of inflammatory mediators and their localization in absence epilepsy.

## Data Availability Statement

The raw data supporting the conclusions of this article will be made available by the authors, without undue reservation, to any qualified researcher.

## Ethics Statement

The animal study was reviewed and approved by Ondokuz Mayıs University Animal Experiments Local Ethics Committee (2015/56).

## Author Contributions

EA planned and supervised all experiments. ED, HA, GA, and MA conducted the experiments. BA and ER provided biochemical measurements. ED, HA, GA, and BA analyzed the data. EA wrote the manuscript.

## Conflict of Interest

The authors declare that the research was conducted in the absence of any commercial or financial relationships that could be construed as a potential conflict of interest.
